# Emricasan for the treatment of liver cirrhosis: a meta-analysis of randomized controlled trials

**DOI:** 10.4314/ahs.v23i2.46

**Published:** 2023-06

**Authors:** Yao Jie, Lei Youchun, Chen Jingxiang

**Affiliations:** 1 Department of Gastroenterology ,The Sixth People's Hospital of Chongqing; 2 Department of Hepatobiliary Pancreatic Surgery, Chongqing Ninth People's Hospital

**Keywords:** Emricasan, liver cirrhosis, randomized controlled trials, meta-analysis

## Abstract

**Introduction:**

The efficacy of emricasan for liver cirrhosis remains controversial. We conduct a systematic review and meta-analysis to explore the influence of emricasan versus placebo on the treatment of liver cirrhosis.

**Methods:**

We have searched PubMed, EMbase, Web of science, EBSCO, and Cochrane library databases through August 2021 and included randomized controlled trials (RCTs) assessing the efficacy of emricasan versus placebo for liver cirrhosis. This meta-analysis was performed using the random-effect model.

**Results:**

Four RCTs were included in the meta-analysis. Overall, compared with control group for liver cirrhosis, emricasan treatment had no substantial impact on MELD (SMD=-0.19; 95% CI=-0.44 to 0.06; P=0.14), INR (SMD=0.12; 95% CI=-0.13 to 0.37; P=0.36), total bilirubin (SMD=-0.27; 95% CI=-0.56 to 0.01; P=0.06), serum albumin (SMD=0; 95% CI=-0.25 to 0.25; P=1.00) or adverse events (OR=1.35; 95% CI=0.56 to 3.24; P=0.50).

**Conclusions:**

Emricasan treatment provided no benefit for the treatment of liver cirrhosis.

## Introduction

Many chronic liver diseases can result in excessive hepatocyte apoptosis, inflammation and fibrosis 1-4. Non-alcoholic steatohepatitis (NASH), hepatitis C, hepatitis B, and alcoholic liver disease are reported to obviously increase apoptosis, which leads to cell repair, inflammation, fibrosis and ultimately cirrhosis 5-8. Caspases, a family of 11 intracellular cysteine proteases, are able to mediate apoptosis and regulate immune responses 9,10.

Inflammatory caspases (e.g. caspases 1, 4, and 5) induce the activation of interleukin-1 family members and initiator caspases (caspases 2, 8, 9, and 10), which have an important role in priming the NLRP3 inflammasome and producing interleukin-1 b 9-12. Thus, inhibition of caspases benefits to alleviate liver inflammation, fibrosis and portal hypertension. Emricasan (IDN-6556), one oral pan-caspase inhibitor, decreased apoptosis, inflammation and fibrosis in animal models of liver injury including NASH and CCl_4_ -induced cirrhosis 13, 14. Emricasan was also reported to decrease excessive caspase activity and alanine aminotransferase (ALT) in patients with hepatitis C and non-alcoholic fatty liver disease 15,16.

However, the benefit of emricasan for liver cirrhosis has not been well established. Recently, several studies on the topic have been published, and the results were conflicting 17-19. With accumulating evidence, wherefore performed this meta-analysis of RCTs to explore the efficacy of emricasan versus placebo for liver cirrhosis.

## Materials and methods

Ethical approval and patient consent were not required because this was a meta-analysis of previously published studies. The meta-analysis was conducted and reported in adherence to PRISMA (Preferred Reporting Items for Systematic Reviews and Meta-Analyses) 20.

### Search strategy, Data extraction and Study eligibility criteria (PICOS)

Two investigators have independently searched the following databases (inception to August 2021): PubMed, EMbase, Web of science, EBSCO and Cochrane library databases. The electronic search strategy was conducted using the following keywords: “liver cirrhosis” OR “hepatic cirrhosis” AND “emricasan”.

We extracted the following information: author, number of patients, age, female, body mass index, total bilirubin and detail methods in each group etc. Data were extracted independently by two investigators, and discrepancies were resolved by consensus. We also contacted the corresponding author to obtain the data when necessary.

PICOS are presented as follows, Participants (P): patients are diagnosed with liver cirrhosis. Intervention (I): emricasan treatment. Control (C): placebo. Outcome (O): The primary outcome is model for end-stage liver disease (MELD) and secondary outcomes include international normalized ratio (INR), total bilirubin, serum albumin and adverse events. Study design (S): RCT

### Quality assessment in individual studies

Methodological quality of the included studies was independently evaluated using the modified Jadad scale 21. There were 3 items for Jadad scale: randomization (0-2 points), blinding (0-2 points), dropouts and withdrawals (0-1 points). The score of Jadad Scale varied from 0 to 5 points. An article with Jadad score≤2 was considered to have low quality. If the Jadad score≥3, the study was thought to have high quality 22.

The risk of bias tool was also used to assess the quality of individual studies in accordance with the Cochrane Handbook for Systematic Reviews of Interventions 23, and the following sources of bias were considered: selection bias, performance bias, attrition bias, detection bias, reporting bias, and other potential sources of bias. The overall risk of bias for each study was evaluated and rated: low, unclear and high.

### Statistical analysis

We estimated the standard mean difference (SMD) with 95% confidence interval (CI) for continuous outcomes (MELD, INR, total bilirubin and serum albumin) and odd ratio (OR) with 95% CI for dichotomous outcomes (adverse events). The random-effects model was used regardless of heterogeneity. Heterogeneity was reported using the I2 statistic, and I2 > 50% indicated significant heterogeneity 24. Whenever significant heterogeneity was present, we searched for potential sources of heterogeneity via omitting one study in turn for the meta-analysis or performing subgroup analysis. All statistical analyses were performed using Review Manager Version 5.3 (The Cochrane Collaboration, Software Update, Oxford, UK).

## Results

### Literature search, study characteristics and quality assessment

**Figure 1:**
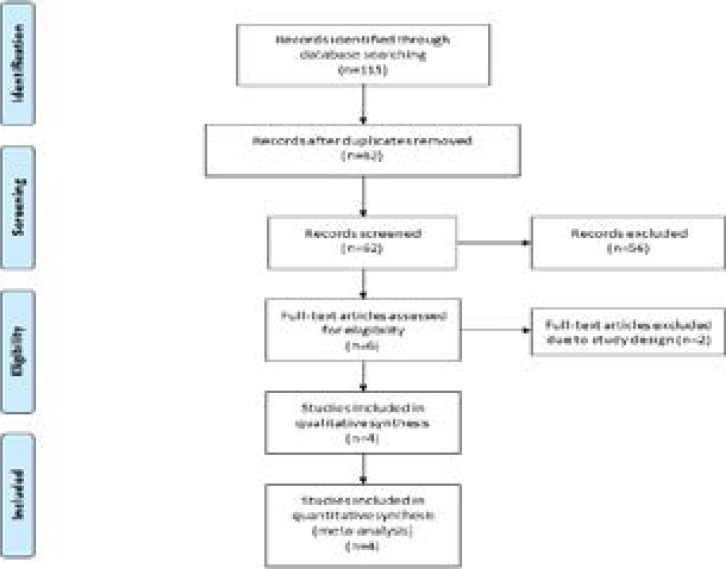
Flow diagram of study searching and selection process

A detailed flowchart of the search and selection results was shown in Figure 1. 115 potentially relevant articles were identified initially and four RCTs were ultimately included in the meta-analysis^17^-^19^, ^25^.

The baseline characteristics of four eligible RCTs were summarized in Table 1. The four studies were published between 2019 and 2021, and total sample size was 423. Emricasan was administered at the dose of 25 mg twice daily, and the treatment duration ranged from 24 weeks to 24 months.

**Table 1:** Characteristics of included studies

NO.	Author	Emricasan group						Control group						Jadad scores
		Number	Age (years)	Female (n)	Body mass index (kg/m2)	Total bilirubin (mg/dL)	Methods	Number	Age (years)	Female (n)	Body mass index (kg/m2)	Total bilirubin (mg/dL)	Methods	
1	Weinberg 2021	41	60.4±8.05	9	28.10±4.330	0.600±0.3392	25 mg twice daily for 24 months	23	61.1±6.09	5	30.51±4.701	0.742±0.4101	placebo	4
2	Frenette 2021	71	60.2±9.80	37	34.3±6.49	1.3±0.71	25 mg twice daily for 24 weeks	70	62.8±6.83	40	33.9±6.95	1.5±0.86	placebo	5
3	Garcia-Tsao 2020	65	62.0±8.8	35	34.4±6.2	-	25 mg twice daily for 24 weeks	67	61.4±7.9	45	35.9±7.9	-	placebo	4
4	Frenette 2019	44	58.5±8.15	20	-	2.25±1.12	25 mg twice daily for 3 months	42	57.5±8.75	12	-	2.59±1.49	placebo	4

**Figure 2:**
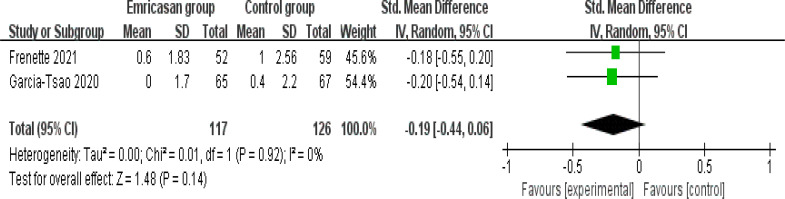
Risk of bias assessment. (A) Authors' judgments about each risk of bias item for each included study. (B) Authors' judgments about each risk of bias item presented as percentages across all included studies

Among four studies included here, two studies reported MELD, INR, total bilirubin and serum albumin [18, 19], as well as two studies reported adverse events [17, 19]. Jadad scores of four included studies varied from 4 to 5, and all four studies were considered to have high quality according to quality assessment. Risk of bias analysis showed that all RCTs generally have high quality (Figure 2).

### Primary outcomes: MELD

**Figure 3:**
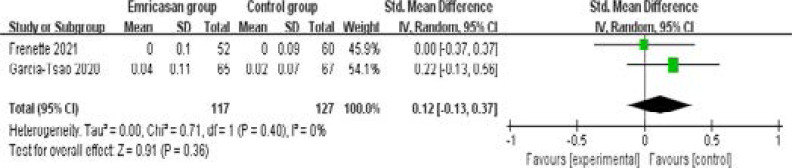
Forest plot for the meta-analysis of model for end-stage liver disease (MELD)

This outcome data was analysed with the random-effects model, and compared to control group for liver cirrhosis, emricasan treatment had no obvious effect on MELD (SMD=-0.19; 95% CI=-0.44 to 0.06; P=0.14) with no heterogeneity among the studies (I2=0%, heterogeneity P=0.92, Figure 3).

### Sensitivity analysis

No heterogeneity was observed among the included studies, and thus we did not perform sensitivity analysis via omitting one study in turn to detect heterogeneity.

Secondary outcomes

**Figure 4:**
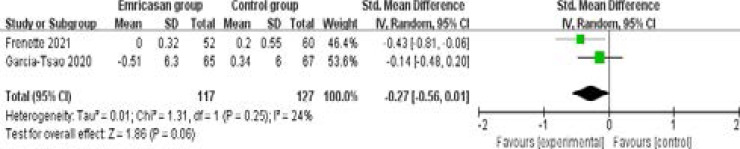
Forest plot for the meta-analysis of international normalized ratio (INR)

**Figure 5:**
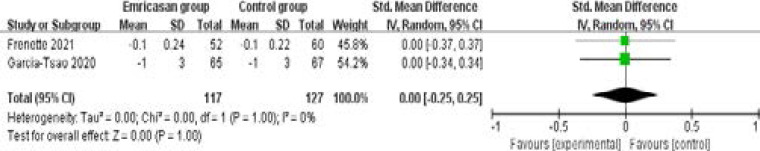
Forest plot for the meta-analysis of total bilirubin

**Figure 6:**
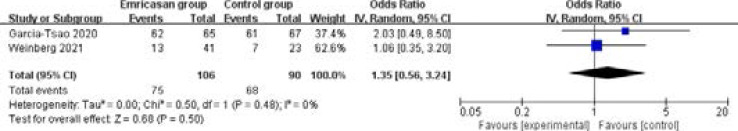
Forest plot for the meta-analysis of serum albumin

**Figure 7:**
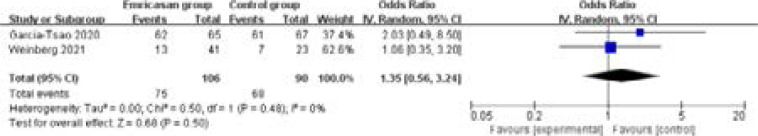
Forest plot for the meta-analysis of adverse events

In comparison with control group for liver cirrhosis, emricasan treatment showed no substantial impact on INR (SMD=0.12; 95% CI=-0.13 to 0.37; P=0.36; Figure 4), total bilirubin (SMD=-0.27; 95% CI=-0.56 to 0.01; P=0.06; Figure 5), serum albumin (SMD=0; 95% CI=-0.25 to 0.25; P=1.00; Figure 6) or adverse events (OR=1.35; 95% CI=0.56 to 3.24; P=0.50; Figure 7).

## Discussion

Cellular injury from lipotoxicity, endoplasmic reticulum stress and viral replication can activate caspases 26-28. Executioner caspases (caspases 3, 6, and 7) are responsible for many cell proteins such as keratin 18 (CK-18), and mediate the production of proinflammatory, profibrotic hepatic microvesicles, which lead to the activation, migration, and profibrotic gene expression by interacting with hepatic stellate/myofibroblasts and sinusoidal endothelial cells 9,29. Emricasan is known as an important caspase inhibitor and has demonstrated the potential in alleviating NASH and CCl4-induced cirrhosis by decreasing apoptosis, inflammation and fibrosis 13, 14.

The benefits of emricasan for hepatic function in subjects with cirrhosis are supported by preclinical studies in the rat CCl4 cirrhosis model, and the potential mechanism is that emricasan is able to decrease intrahepatic vascular resistance and inflammation, improve sinusoidal cell/hepatic stellate cell phenotype and microcirculatory function 14. Emricasan treatment is able to reduce flCK-18 and the executioner caspase 3/7 25.

Our meta-analysis included four RCTs and 423 patients with liver cirrhosis, and the results confirmed that compared to placebo for patients with liver cirrhosis, emricasan treatment showed no improvement in MELD, INR, total bilirubin or serum albumin. These suggested that emricasan had no treatment efficacy for patients with liver cirrhosis. Regarding the sensitivity analysis, no significant heterogeneity remained. However, several factors may produce some bias. Firstly, liver cirrhosis was caused by NASH and hepatitis C 17-19, 25, and different etiologies may affect the efficacy assessment. Secondly, the treatment duration of emricasan ranged from 24 weeks to 24 months, which may produce some bias for the results. Thirdly, liver cirrhosis with different severity levels may have various responses to the emricasan treatment. In addition, the emricasan was safe for these patients based on the results of adverse events in our meta-analysis.

Our meta-analysis also has some important limitations. Firstly, our analysis is based on four RCTs, and two of them have a relatively small sample size (n<100). Overestimation of the treatment effect is more likely in smaller trials compared with larger samples. Although there is no significant heterogeneity, different etiologies and severity levels of liver cirrhosis may produce some bias. Finally, treatment duration ranges from 24 weeks to 24 months, and the duration may be not sufficient to produce the positive results.

## Conclusions

Emricasan treatment may provide no benefits for patients with liver cirrhosis.
